# Adaptive introgression from indicine cattle into white cattle breeds from Central Italy

**DOI:** 10.1038/s41598-020-57880-4

**Published:** 2020-01-28

**Authors:** Mario Barbato, Frank Hailer, Maulik Upadhyay, Marcello Del Corvo, Licia Colli, Riccardo Negrini, Eui-Soo Kim, Richard P. M. A. Crooijmans, Tad Sonstegard, Paolo Ajmone-Marsan

**Affiliations:** 1 Università Cattolica del Sacro Cuore, Department of Animal Science Food and Nutrition – DIANA, Nutrigenomics and Proteomics Research Centre – PRONUTRIGEN, and Biodiversity and Ancient DNA Research Centre, Università Cattolica del Sacro Cuore, Piacenza, Italy; 2 School of Biosciences, Cardiff University, Cardiff, Wales UK; 3 Animal Breeding and Genomics, Wageningen University & Research, Wageningen, The Netherlands; 4 Department of Animal Breeding and Genetics, Swedish University of Agricultural Sciences, Uppsala, Sweden; 5 Acceligen, Recombinetics, Eagan, MN USA

**Keywords:** Genetic hybridization, Genomics, Genotype, Population genetics

## Abstract

Cattle domestication occurred at least twice independently and gave rise to the modern taurine and indicine cattle breeds. European cattle diversity is generally dominated by taurine cattle, although elevated levels of indicine ancestry have been recorded in several breeds from southern Europe. Here we use genome-wide high-density SNP genotyping data to investigate the taurine and indicine ancestry in southern European cattle, based on a dataset comprising 508 individuals from 23 cattle breeds of taurine, indicine and mixed ancestry, including three breeds from Central Italy known to exhibit the highest levels of indicine introgression among southern European breeds. Based on local genomic ancestry analyses, we reconstruct taurine and indicine ancestry genome-wide and along chromosomes. We scrutinise local genomic introgression signals and identify genomic regions that have introgressed from indicine into taurine cattle under positive selection, harbouring genes with functions related to body size and feed efficiency. These findings suggest that indicine-derived traits helped enhance Central Italian cattle through adaptive introgression. The identified genes could provide genomic targets for selection for improved cattle performance. Our findings elucidate the key role of adaptive introgression in shaping the phenotypic features of modern cattle, aided by cultural and livestock exchange among historic human societies.

## Introduction

Livestock domestication is tightly intertwined with the evolution and welfare of human societies, contributing to the transition from a nomadic to sedentary lifestyle^1–4^. Understanding the origin, dispersal and evolution of globally important livestock species such as cattle and sheep has thus been the focus of numerous genetic investigations (e.g.^5–7^). European taurine cattle (*Bos taurus*) are believed to first have been domesticated from *Bos primigenius primigenius* in the Fertile Crescent ~10,000 years ago (YA)^8,9^. A second inferred domestication event occurred about 2,000 years later in the Indus valley, Central Asia, with *Bos primigenius namadicus* as ancestor of humped zebu cattle (*Bos indicus*)^10,11^. Following the expansion of agriculture, taurine cattle colonized Europe, Asia and Africa, and reached Southern Europe around 8,000 YA^12^. Similarly, indicine cattle were taken along by humans across South-East Asia, and later on into Africa^13^. The current geographical distribution of Eurasian cattle breed ancestry reflects these migration paths, with taurine cattle populating Europe and Northern Asia, and indicine Southern Asia, respectively^14–18^, and different degrees of admixture of taurine and indicine lineages giving rise to African taurine, African zebu, and their crosses known as Sanga cattle^19,20^.

Genome-wide analyses have provided evidence that several southern European breeds contain ancestry from both African taurine and indicine lineages^15,21^. One example are the geographically widespread Podolian cattle breeds^22^, whose name refers to their presumed origin in Moldova/Western Ukraine. Among Podolian cattle are some breeds from Central Italy which display phenotypes such as red coat in calves and white to light grey coat in adults, and a predisposition for draught and beef production, all typical traits of Podolian cattle breeds^22^. Such breeds – often referred to as Central Italian white cattle – also show the highest levels of indicine ancestry among southern European cattle^12,15,21^.

Indicine cattle are known to perform better than taurine at harsher conditions (e.g., feed- and heat-stress, resistance to parasites and diseases, drought^23–25^). To date, however, no assessment of the putative adaptive potential of the indicine-derived genomic component present in southern European cattle breeds has ever been undertaken.

Recent technological advances allow affordable use of high-density DNA arrays able to scan several thousand genome-wide markers for all of the major livestock species^26,27^. Such information has been used in several studies to successfully identify fine-scale gene-flow, selection signatures and association of allelic variants to quantitative traits in a variety of livestock species^28–30^. The acquisition of fitness-enhancing regions due to gene-flow – as the consequence of either natural or human-mediated events – is referred to as adaptive introgression^31^. Dense genome-wide information allowed the development of local ancestry inference (LAI) approaches able to assign ancestry along a chromosome and identify ancestry blocks^32^. Hence, adaptive introgression can be identified by LAI, by detecting those regions of fixed or nearly-fixed genome ancestry from a specific source population^33^. Currently, LAI has been mostly performed on high-density human datasets^32–35^, and medium-density DNA array data in canids and ovines^36–38^.

Here we survey the genomes of three autochthonous white cattle breeds from Central Italy, Chianina, Romagnola, and Marchigiana. We study the indicine-derived introgression in these Italian white cattle breeds using genome-wide HD SNP array, combining LAI and selection signature analysis to pinpoint such introgression at the chromosome level. We then infer the adaptive potential of the introgressed genomic regions, along with their putative geographical origin.

## Materials and Methods

BovineHD Genotyping BeadChip (777k SNPs) genotypes from 501 individuals across 16 cattle breeds representing *B. taurus* and *B. indicus* were used (Table 1). Blood samples from 16 Chianina, 13 Marchigiana and 30 Romagnola individuals were provided by ANABIC ‘Associazione Nazionale Allevatori Bovini Italiani Carne’ (http://www.anabic.it/). Samples were collected by veterinarians between the years 2002–2011, complying with the EU Directive 86/609/EEC regulations which did not require the approval of animal welfare/ethics committee and with the agreement of breeders. The GenElute Mammalian Genomic DNA Miniprep Kit (Sigma-Aldrich) was used for total DNA extraction following the manufacturer’s instructions. Genotypes were produced at Laboratorio Genetica e Servizi (LGS) Cremona, Italy. BovineHD genotypes of the other 13 taurine and indicine breeds were kindly made available by Tad Sonstegard (previously unpublished data). BovineHD genotypes of seven additional breeds from Iberia, Italy, and Eastern Europe were available from Upadhyay *et al*.^14,39^ (Table 1).

**Table 1:** Sample information and diversity indices. Diversity indexes were computed for those breeds having sample size >10).

Acronym	Breed	Origin	N	H_o_ (SD)	N_e_	F_ROH_ (SD)
**B. taurus europeus (European taurine)**						
ANG	Angus	European	37	0.29 (0.19)	135	0.29 (0.04)
BSW	Brown Swiss	European	54	0.29 (0.20)	98	0.27 (0.03)
FLV	Fleckvieh	European	55	0.30 (0.19)	126	0.22 (0.02)
HFD	Hereford	European	24	0.30 (0.18)	118	0.34 (0.09)
HOL	Holstein	European	55	0.31 (0.19)	110	0.24 (0.03)
LMS	Limousin	European	40	0.31 (0.19)	152	0.21 (0.02)
PIE	Piedmontese	North Italy	24	0.32 (0.19)	147	0.17 (0.01)
MCG	Marchigiana	Central Italian White Cattle	13	0.31 (0.21)	107	0.20 (0.03)
ROM	Romagnola	Central Italian White Cattle	30	0.30 (0.19)	119	0.22 (0.02)
CHI	Chianina	Central Italian White Cattle	16	0.30 (0.21)	116	0.24 (0.01)
MAM	Maremmana	Central Italian	5	—	—	—
POD	Podolica	Southern Italian	1	—	—	—
BUS	Busha	Eastern European	6	—	—	—
RUG	Romanian Grey	Eastern European	4	—	—	—
BOK	Boskarin	Eastern European	4	—	—	—
CAR	Cardena	Iberian	5	—	—	—
LID	Lidia	Iberian	3	—	—	—
**B. taurus africanus (African taurine)**						
NDA	N’Dama	West Africa	48	0.24 (0.18)	148	0.31 (0.08)
**Sanga (African admixed taurine/indicine)**						
ANW	Ankole-Watussi	Central Africa	25	0.30 (0.19)	175	0.20 (0.01)
NGA	Nganda	Central Africa	26	0.32 (0.19)	120	0.17 (0.04)
**B. indicus**						
GIR	Gir	India	28	0.21 (0.20)	180	0.30 (0.03)
LOH	Lohani	India/Pakistan	13	0.20 (0.22)	104	0.32 (0.07)
THA	Tharparkar	Pakistan	13	0.19 (0.23)	63	0.39 (0.07)
total			529			

N, number of individuals analysed in this work; Ho, observed heterozygosity and its standard deviation (SD); Ne, effective population size inferred 13 generations in the past; FROH, inbreeding coefficient computed from Runs of Homozygosity and its standard deviation (SD).

SNP pruning of genotype data was performed using PLINK v1.7^40^. Loci having a minor allele frequency <0.01 and call rate <0.9 were removed. Data were phased using SHAPEIT v2.r790^41^. SNPs located on sex chromosomes or with unknown map positions were removed. Linkage disequilibrium pruning was performed using the ‘–indep-pairwise’ function in PLINK, where SNPs with *r*^2^ > 0.25 were removed from sliding windows of 50 SNPs and a step size of five SNPs.

### Summary stats and genetic structure

Observed heterozygosity values were calculated using custom scripts. Inbreeding levels for each population (*F*_*ROH*_) were computed as the average of the individual proportion of Runs of Homozygosity (ROHs) to the total length of the genome; ROHs were computed using PLINK. Effective population size (*N*_*e*_) was estimated using SNeP v1.11^42^ applying sample size correction for phased genotypes, and Sved & Feldman’s mutation rate modifier which performs well in inferring *N*_*e*_ for the most recent generations^43^.

A Neighbour-Net graph using Reynolds’ distances, computed with a custom script, was generated using SplitsTree v4.14.6^44^. Maximum likelihood analysis of population structure was conducted using Admixture v1.3.0^45^ for *K* values from 2 to 16, the latter corresponding to the total number of breeds sampled in our study. A principal component analysis (PCA) was performed to investigate the ordinal relationships between populations and individuals, using PLINK.

### Analysis of local genomic ancestry

We used PCAdmix v1.0^46^ to infer genomic local ancestry. For each chromosome and haploid individual, this software scans the target genome using a sliding-window approach, determining the relative ancestry proportions to the utilised reference populations. Window size depends on genome density; here default parameters were used (20 SNPs per window) as the optimal marker density suggested by the software authors matches the BovineHD SNPChip marker density (~1 SNP every 3 kb).

Local ancestry inference relies on reference populations representative of the putative ancestral populations that contributed to the current genomic composition in the target genome. Reference choice is key, along with sufficiently dense data along the chromosomes, to allow reliable ancestry inference^37,46^. In practical terms, reference choice is tricky, as recent demographic histories of the reference breeds can lead to confounding results^46^. An approach to partially overcome such issue requires to i) perform multiple analyses with different populations/breeds as representative of a same reference, ii) and then pool the results and identify consensus signals of ancestry, as implemented in the ‘Consistently Introgressed Windows of Interest’ (CIWI) framework^37^.

**Figure 1:**
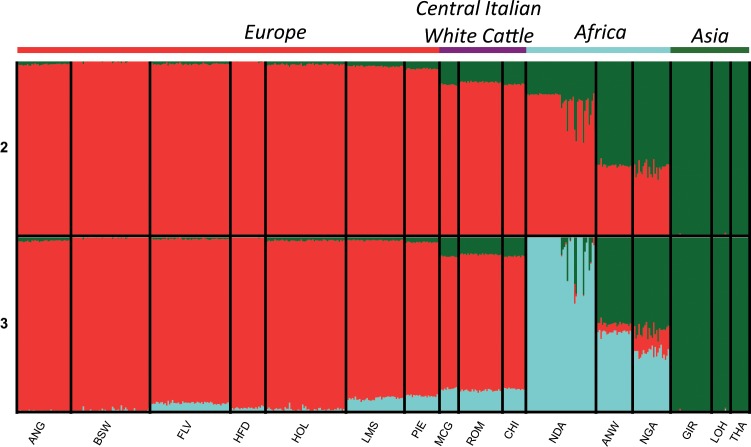
Admixture plot comprising clustering solutions (*K* = 2–3) for 16 cattle breeds, based on 647,132 SNPs from the BovineHD SNPChip. The geographical origin of the breeds is indicated above the plot. Breed labels are available in Table 1. Results for higher values of *K* are shown in Fig. S1a.

To identify reference-independent signals of introgression we applied the CIWI-framework using Chianina, Marchigiana, and Romagnola as target populations. Nine LAI analyses were performed for each target population using as reference breeds all the possible pairwise combinations of one taurine breed among Hereford, Fleckvieh, and Brown Swiss, and one indicine breed among Tharparkar, Gir, and Lohani. These reference breeds were chosen among those not showing mixed cluster components according to *K* = 2 from the Admixture analysis (Fig. 1). To reduce the possible bias due to productive trait selection, breeds selected for different productive purposes (milk, meat, and dual-purpose) were chosen.

To investigate the putative Eastern or Western origin of the main introgression signals, additional LAI tests focusing on chromosomes 7 and 18 (BTA7 and BTA18, respectively) were performed on genotype data of seven additional breeds, including two cattle breeds from the Iberian peninsula (Cardena and Lidia), two from Central and Southern Italy (Maremmana and Podolica), and three from Eastern Europe and Balkans region (Busha, Romanian grey and Boskarin; Table 1). These genotype data were merged with data used in the previous analysis and submitted to LAI using the same set of nine reference combinations as previously described. To further investigate the origin of BTA7 and BTA18 introgression, these regions were tested in Chianina using PCAdmix/CIWIs with the three indicine references (Tharparkar, Lohani, Gir), and one African taurine reference provided by 25 non-admixed N’Dama individuals (Fig. 1).

### Selection signatures

Introgressed genomic portions are kept by selection if provide fitness enhancing variants. Hence, it is likely for a genomic region with adaptive significance to host a selective sweep. Here, we tested the presence of signatures of selection using the haplotype-based statistic *nSL*^47^ as implemented in the software Selscan v1.2.0a^48^. This approach uses the decay of haplotype homozygosity as a function of recombination distance to detect selection sweeps in genomic data^47^. Further, *nSL* is well suited to detect selection in loci at low-medium frequency, as in incomplete sweeps on standing variation^47,49^. Ancestral allele information was available from Rocha and colleagues^50^. Selection signature analysis was performed on Chianina BTA18 and BTA7. Results were first normalised, and then smoothed by means of cubic smoothed spline using R^51^.

## Results

### Genetic diversity and structure

After SNP pruning, 647,132 loci of the BovineHD SNP array were left for analysis. Observed heterozygosity ranged from 0.29 to 0.32 for European taurine breeds and the two Central African Sanga (Ankole-Watussi, Nganda), whereas the Central African N’Dama recorded 0.24. The three indicine breeds (Tharparkar, Lohani and Gir) recorded the lowest *H*_*o*_ values (0.19–0.22; Table 1).

Current *N*_*e*_ estimates from SNeP for European taurine breeds ranged between 98 and 152 (Table 1), with Brown Swiss showing the lowest (98), and Limousine and Piedmontese the highest values (138 and 144, respectively). African taurine recorded similar values (range 120–175). Among indicine cattle Gir recorded the highest *N*_*e*_ estimates (180), and Tharparkar the lowest (63; Table 1). Inbreeding values for European taurine ranged 0.17 (Piedmontese) to 0.34 (Hereford), similarly to the African breeds (0.17–0.31). The indicine population recorded higher inbreeding values on average (0.30–0.39), with highest values found in Tharparkar (Table 1).

In the Admixture analysis, estimates of CV decreased with increasing values of *K* from 1 to 16, with the main separation between *B. taurus* and *B. indicus* at *K* = 2 (Supplementary Fig. S1b). Admixture analysis at *K* = 2 split the dataset into three groups showing taurine, indicine and taurine *x* indicine ancestries (Fig. 1). These three groups overlapped with the European, Southeast Asian, and African origin of the breeds, respectively (Fig. 1, Supplementary Figs. S1a and S2). While most European breeds showed >97% taurine ancestry, approximately ~11–13% of indicine ancestry were recorded in three Central Italian breeds (Marchigiana, Romagnola, and Chianina). The three indicine breeds in our dataset grouped together at this clustering level. At *K* = 3 the African taurine component was identified; this cluster was found in African taurine N’Dama and comprised 30–47% of total ancestry of the two Sanga breeds Ankole-Watussi and Nganda (Fig. 1). Several African taurine N’Dama individuals show high levels of indicine ancestry, probably reflective of recent crossbreeding with indicine or indicine crossbreds. As found previously, levels of African taurine ancestry were identified in all of the southern European breeds: Fleckvieh (a Simmental-derived breed; ~5%), Limousine from central France (~8%), and Piedmontese from North-West Italy (~10%); the largest values were recorded in the three cattle breeds from Central Italy (Chianina, Marchigiana and Romagnola; range ~11–14%; Fig. 1).

In the PCA (Supplementary Fig. S6), the first principal component (PC) accounted for 18.5% of the variance and discriminated taurine and indicine cattle, mirroring the Admixture results obtained at K = 2. Similarly the second PC accounted for 4.4% of the variance and reflected Admixture results for K = 3, discriminating African taurines from other populations.

### Local ancestry inference

**Figure 2:**
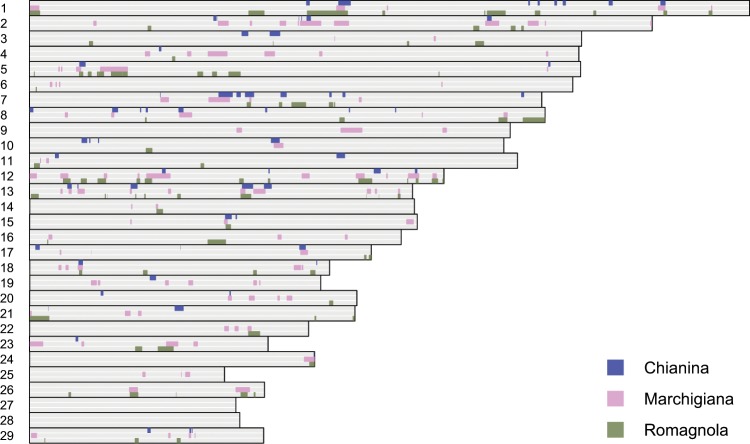
Consistently Introgressed Windows of Interest (CIWIs) in three Central Italian white cattle breeds (Chianina, Marchigiana and Romagnola), identifying genomic regions of indicine-derived ancestry. Each grey horizontal bar corresponds to one cattle autosome (1–29), and local ancestry within each of the three breeds is shown in one row per breed along each chromosome. Indicine ancestry, when evidenced, is shown in blue, pink and green, respectively, for each breed. Grey indicates a lack of consistent evidence of indicine ancestry.

**Figure 3:**
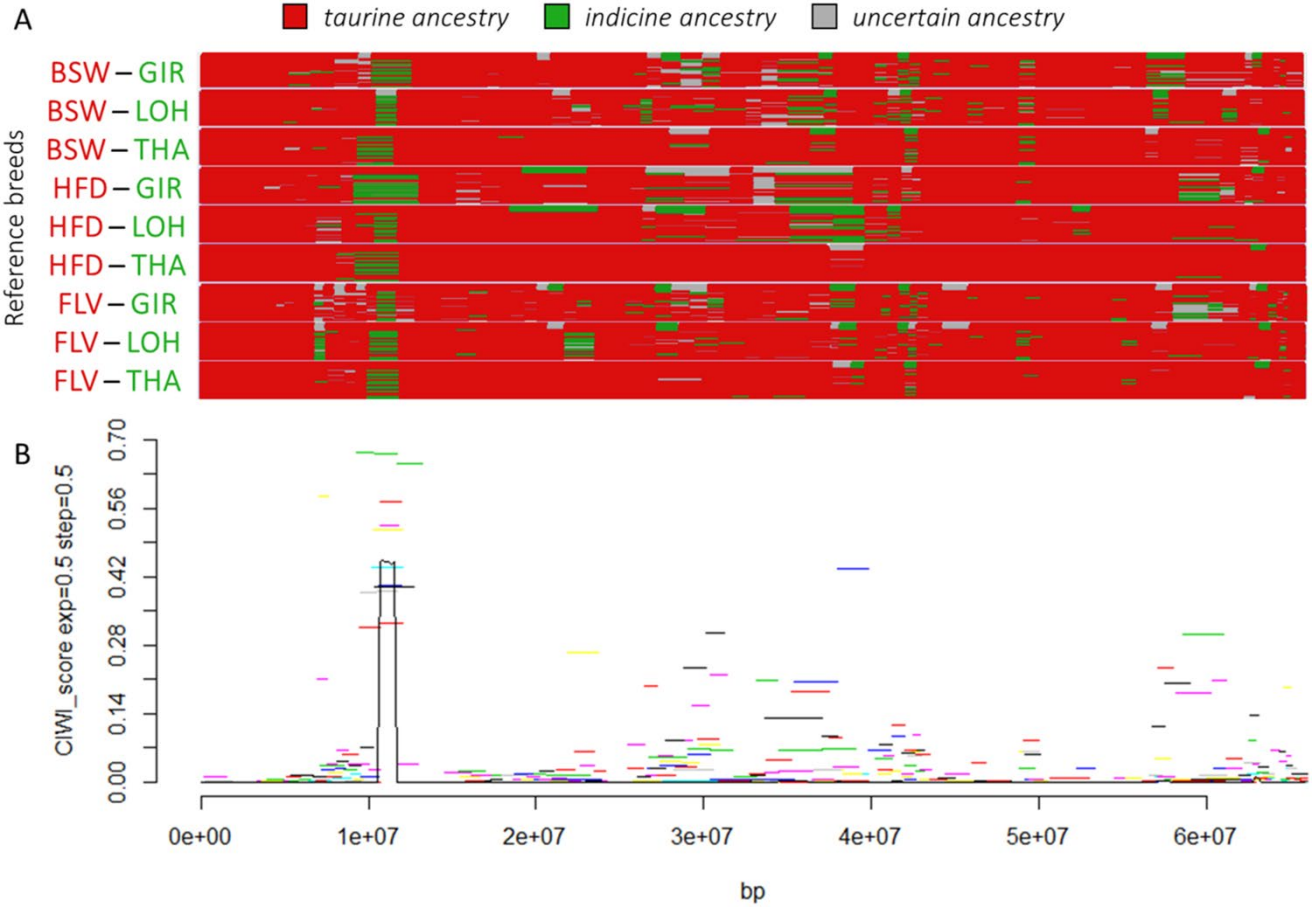
Local ancestry signals of indicine introgression into taurine cattle breeds, identified from chromosome painting, and identified Consistently Introgressed Windows of Interest (CIWIs) on BTA18. (**A**) Graphical representation of the chromosome painting results obtained using different combinations of reference populations of both taurine (red) and indicine (green) ancestry. (**B**) CIWI results for the same chromosome. See Table 1 for breed labels.

We explored the relevant levels of indicine component in Chianina, Romagnola, and Marchigiana by means of local ancestry inference, comparing these three breeds with nine reference combinations and applied the CIWIs framework to aggregate PCAdmix results (Figs. 2 and 3). We identified eight regions of indicine-derived ancestry shared by the Central Italian white-cattle breeds within the top 5% of the genome-wide CIWI scores (Supplementary Table S1). Within these genomic regions, we identified 25 genes spread across seven chromosomes (1, 5, 7, 13, 15, 18, and 24; Table 2). More stringently, 24 of these genes were found within the top 1% of CIWI scores in at least one of the three breeds, but none of them shared by all the three breeds (Table 2).

**Table 2:** Genes located within the top 5% CIWIs shared by three Central Italian white cattle breeds, based on BovineHD SNPChip data. See Supplementary Table S1 for the full list, and Table 1 for breed labels.

Chr	Position	Gene ID	Associated function	Top 1%*		
				CHI	MCG	ROM
1	68.01–68.10	PDIA5				x
1	68.26–68.42	ADCY5				x
1	68.46–68.55	HACD2				x
1	68.90–68.94	CCDC14	Beef traits			x
1	68.95–68.99	ROPN1	Beef traits			x
1	139.68–139.84	CPNE4	Growth traits		x	
5	11.31–11.31	TMA7				
7	48.39–48.47	H2AFY	Muscle regeneration	x		x
7	48.62–48.62	NEUROG1		x		x
7	48.66–48.67	CXCL14	Body weight	x		x
13	46.78–46.83	LARP4B				x
13	47.04–47.18	DIP2C				x
13	47.20–47.26	ZMYND11				x
13	47.40–47.42	PRNP				x
13	47.44–47.45	PRND				x
15	42.97–43.48	SBF2	Growth traits		x	x
15	43.51–43.59	SWAP70			x	x
18	10.88–10.94	USP10	Gluconeogenesis	x		
18	10.99–11.05	CRISPLD2	Residual feed intake	x		
18	11.12–11.13	ZDHHC7	Residual feed intake	x		
18	11.17–11.19	KIAA0513	Residual feed intake	x		
18	11.20–11.21	FAM92B	Residual feed intake	x		
24	62.51–62.53	SERPINB7				x
24	62.59–62.61	SERPINB2				x
24	62.62–62.64	SERPINB10	Feed efficiency			x

Chr, chromosome; Position, physical coordinates in Mb. *While all genes listed in the table are in the top 5%, top 1% indicates which genes were recorded at an even higher stringency threshold (1%) applied on the genome-wide CIWI results.

The strongest CIWI signal was recorded on BTA18 between 10.80 and 11.74 Mb (CW-18), with the highest values recorded in Chianina (Supplementary Fig. S5). Five genes: *USP10*, *CRISPLD2*, *ZDHHC7*, *KIAA0513*, and *FAM92B* (Table 2), were identified within the top 5% selection in all the three Central Italian cattle breeds. With the exclusion of *USP10* all of these genes have previously been associated with residual feed intake (RFI) in indicine cattle^52^.

The second strongest CIWI signal was recorded in BTA7 between 47.75 and 49.38 Mb (spanning ~1.6 Mb; hereafter called CW-7). Out of the 18 genes included within this genomic region only three (*H2AFY*, *NEUROG1*, and *CXCL14*) were shared by all the Central Italian cattle breeds in the top 5% selection (Table 2). *CXCL14* has been associated to body weight in cattle^53^. No direct phenotypic associations have been proposed for *H2AFY* in cattle, but splicing dysregulation of this gene has been associated with skeletal muscle regeneration disorders in murine models^54^. Further, the majority of the genes included in the CIWI – although below the top 5% threshold – have been directly associated to stature (*CAMLG*, *DDX46*, *TXNDC15*, *CATSPER3*, *PITX1*^55^), body weight (*SLC25A48*, *FBXL21*^53^), and cartilage and body weight development/differentiation (*LECT2*, *TGFBI*^56^) in cattle.

Among the 24 genes also identified within the top 1% CIWI (Table 2), are *CCDC14*, *ROPN1*, and *CPNE4* (BTA1), *SBF2* (BTA15), and *SERPINB10* (BTA24). The differential expression of *CCDC14* and *ROPN1* have been related to beef traits^57,58^, whereas *CPNE4* and *SBF2* have been associated with growth traits in horse and cattle, respectively^59,60^. *SERPINB10* is involved in the amino acid metabolism and has been associated to feed efficiency traits in ruminants^61^.

Using seven additional breeds from Italy, the Balkan area, and Iberia (Table 1), we next performed additional CIWI analyses focused on the two main signals (CW-7 and CW-18) we identified using the BovineHD SNP panel (see above). Both genomic regions were identified as putatively introgressed in Maremmana and Podolica. Among the East European breeds, CW-7 was again identified in Busha and Romanian grey, whereas Boskarin did not show signal for either CW-7 or CW-18. Similarly, no signal was detected corresponding to CW-7 and CW-18 among the Iberian breeds. We then tested the hypothesis of a possible African ancestry for these two signals using the PCAdmix/CIWI framework with both African taurine and indicine breeds as reference and targeting Chianina. For both genomic regions, we recovered a signal of indicine (rather than African taurine) ancestry, overlapping with CW-18, and a partial overlap with the CW-7 (Supplementary Fig. S3).

### Signatures of selection

Using *nSL*, we investigated the presence of signature of selection co-occurring in the genomic portions identified using the CIWIs approach to further test the adaptive significance of these putatively introgressed genomic regions. Indeed, one of the highest scoring selection sweeps perfectly overlapped CW-18 (Supplementary Fig. S4). Further, selection signals overlapped with CIWI signals identified in BTA1 and BTA24 (Supplementary Fig. S4). Conversely, we found no evidence of any marked selective sweep overlapping CIWI regions identified in BTA5, BTA7, BTA13, and BTA15 (Supplementary Fig. S4).

## Discussion

We investigated patterns of local ancestry in several cattle breeds from Central Italy using high-density genome-wide polymorphism data. We identified signals of indicine ancestry at genes that in indicine breeds are associated with feeding efficiency. Further, we studied the occurrence of the same signals in several breeds from East and West Europe. Our results suggest adaptive introgression of indicine-derived alleles into Central Italian white cattle breeds, possibly as the result of several gene flow events.

### Admixture between taurine and indicine cattle

Population structure analysis (Figs. 1 and 2) performed on genotype data identified >10% indicine derived ancestry in the white cattle breeds from Central Italy, and signals of 5–14% African taurine ancestry in several breeds from southern Europe - consistent with previous findings^9,15,21^. The three autochthonous Italian breeds remained in a joint Admixture cluster until *K* = 4, but at higher values of *K* the divergence of Romagnola from Chianina and Marchigiana is apparent (Supplementary Figs. S1a and S6). Among the three, Chianina is the only breed with no available accounts of introgressive crossbreeding. For Romagnola and Marchigiana, however, records from the 19^th^ century describe the use of Chianina bulls to improve draught and body size traits of both breeds^62^. In addition, crosses between Marchigiana and Romagnola bulls were later performed to decrease the large Marchigiana body size which – after being crossed with Chianina – was too large and unsuited for grazing hilly pastures^62^. This documented admixture may explain the extensive shared ancestry of Chianina and Marchigiana at *K* = 13, and the smaller component of Romagnola ancestry in Marchigiana (Supplementary Fig. S1a). Our obtained clustering results therefore confirm a complex history of cross-breeding and enhancement along with strong selective breeding in these Italian cattle breeds.

In our dataset, genetic diversity of indicine breeds appears lower than that of European breeds (Table 1). This pattern contrasts with the documented reduced effective population size within the highly managed European breeds, and with previously published microsatellite and sequence data from other Asian indicine breeds^10,63^. The demographic patterns (e.g., bottlenecks) of our sampled indicine populations could in principle explain this discrepancy. However, although some indicine breeds were included in the BovineHD SNP array ascertainment panel, it is likely that our diversity results were impacted by ascertainment bias^26^. Hence, we caution that our analyses might underestimate the magnitude of indicine-derived alleles in southern European taurine breeds. Importantly, ascertainment bias impacts frequency-based statistics to a larger extent than multi-locus (e.g., Admixture) and haplotype-based (e.g., PCAdmix) statistics^64–67^. Regardless, our analysis suggest that southern European cattle show significant admixture levels with both indicine and African taurine cattle. Whether this is the result of one or several independent admixture event will require more extensive sampling of cattle breeds, and likely involve analysis of ancient DNA.

### Signals of adaptive introgression

Chianina, Romagnola, and Marchigiana cattle were historically used mostly for draught, and coped well in rough terrain and poor quality pastures. Nowadays, these breeds are highly valued for their meat (e.g., Chianina and Marchigiana T-bones are used for the ‘Fiorentina steak’) and therefore subject to higher management standards to maximise production, but retained both hardiness and rusticity^62^. Our analyses identified multiple regions of putative indicine derived ancestry in Central Italian taurine cattle (Fig. 2), with the strongest signals recorded on BTA18 and BTA7 (Supplementary Fig. S5).

The genomic region CW-18 (~1 Mb) showed the highest CIWI signal genome-wide (Supplementary Fig. S5). Using a haplotype-based selection analysis (Supplementary Fig. S4), we identified four putative genes (*KLHL36*, *USP10*, *KIAA0513*, and *FAM92B*) which appear to have been the target of positive selection. *KLHL36* belongs to the Kelch superfamily, which consists of a large number of structurally and functionally diverse proteins characterized by the presence of a Kelch-repeat domain^68^. Currently, no specific studies are available explaining the role of Kelch proteins on cattle physiology. However, studies on human data led to the identification of Kelch family members as regulators of skeletal muscle development and function^68^. *USP10* has been suggested to have a role in regulating gluconeogenesis^69^, a metabolic pathway involved in the formation of glucose from non-carbohydrate precursors. In non-ruminant species this process is mostly activated during fasting, starvation, and more in general when no exogenous glucose is provided. Conversely, gluconeogenesis in ruminants occurs continuously as dietary carbohydrates are mostly metabolised in the rumen, and gluconeogenesis provides up to 90% of the glucose required^70^. To date, the involvement of *USP10* in gluconeogenesis has only been recorded in pigs – a monogastric species. However, our findings suggest *USP10* as a putative candidate for specific investigations on the feed efficiency of ruminants. *KIAA0513* and *FAM92B* were among those genes identified in Chianina when the top 1% threshold was applied for CIWIs selection (Table 2). Both genes are part of a four gene cluster associated with RFI^52^. RFI is defined as the difference between actual feed intake and the feed intake required to meet maintenance requirements and growth, and reflects the ability of an animal to process food more efficiently and consequently thrive on poor fodder^52,71^. RFI seems to be a polygenic trait in both taurine and indicine cattle^72^. Importantly, indicine cattle are known for the good utilisation of low-quality fodder, higher growth rates, and lower weight losses during droughts^13,73,74^. Selection analysis confirmed that CW-18 harbours a selective sweep, hence we infer that the indicine-derived region CW-18 was favoured by positive selection, putatively allowing for more efficient consumption of poor quality fodder in Central Italian white cattle. Higher feed efficiency has likely been an advantageous trait in the past, especially during harsh environmental conditions with restricted food availability. However, feed efficiency in terms of improved ability to metabolize nutrients is still a highly desirable characteristic for cattle breeders as feed accounts for approximately 70–90% of the total cost in animal production, along with the inherent environmental cost of feed production. Hence, animals that use feed more efficiently positively affect both the costs and sustainability of beef production systems^75,76^. Furthermore, lower RFI in ruminants is also coupled with reduced generation of methane^77^, positively affecting greenhouse gas production, a critical parameter with regard to current climate change^78,79^.

We identified CW-7 (~1.6 Mb) as the second strongest signal of indicine-derived introgression. Signature of selection analysis did not show any evident selective sweep in CW-7 (Supplementary Fig. S4). However, signals for positive selection have previously been identified for the same genomic region in Pingzauer^80^. Within this region several genes are mapped related to body size, and muscle and bone development along with the growth-related *CPNE4* and *SBF2* genes we identified in BTA1 and BTA15, the former overlapping a signature of selection signal. It is possible that CW-7 alleles contribute to the increased body size in some cattle breeds, which is likely under positive selection for draught. Additionally, historical records dating back to Roman times mention the presence of thick-set and powerful cattle in Etruria and Latium and the use of white large cattle in ritual parades since Roman times (Columella, De Re Rustica, VI, 1–3^62,81^). Our CW-7 results may thus mirror this anecdotal evidence. Overall, zebus tend to have longer legs and a more slender morphology than taurine cattle^82,83^. Hence, it is possible that the use of Chianina ancestors in public displays as ritual animals might have been one of the factors contributing to Chianina stature, as both large and tall animals were probably preferred and selected. Morphology has a relevant effect on the ability to adapt to different climates. In indicine cattle, high stature and relatively low body transversal diameters contribute to increase the body-surface/volume proportion, and together with dewlap, large ears and abundant and thin skin, to heat dissipation^84^. According to archaeological records at the time cattle reached southern Europe - and until 6,000–4,000 YA - the Mediterranean climate was hotter and drier^85^. Hence, it is possible that Chianina large but slender body type provided a combination of draught, elegance, and heat-resistance features which over time promoted and maintained its morphology.

### Inference on the origin of the introgression

Using CW-18 and CW-7 as markers in several Iberian and Eastern European breeds we traced the origin of the indicine-derived components in the Central Italian white cattle breeds. Our results lend support to the hypothesis of multiple routes of introgression sourcing from the East. The strong signal we recorded for CW-18 was common to all the analysed Italian breeds (Chianina, Marchigiana, Romagnola, Maremmana, and Podolica), but was not present in any of the Iberian (Lidia and Cardena) or Eastern European breeds (Busha, Romanian Grey, and Boskarin). Conversely, CW-7 was recorded in all the Italian breeds but Romagnola, in two of the East European breeds, but not in the Iberian breeds. Further, local ancestry analysis assigned indicine rather than African taurine ancestry to both genomic regions (Supplementary Fig. S3). It is therefore possible that these two distinct patterns are reflective of at least two different introduction events from the Near East. Indeed, mitochondrial data suggest genetic proximity between central Italian and Podolian cattle^22^, and the CW-7 signal could be the consequence of gene-flow between indicine cattle and the cattle populations on the Podolian plateau, which subsequently spread towards southern Europe^86^. While the CW-18 signal identified in the Central Italian cattle breeds might result from a separate migration route (e.g., via a sea migration from the eastern Mediterranean region ~3,000 YA as suggested by mitochondrial analysis, or following the silk road route 2,000 years later^87,88^), single admixture scenarios coupled with founder effects could also explain the obtained results. Further investigations with more extensive sampling of local cattle populations will therefore be required to firmly assess these scenarios.

The eastern origin of indicine-derived ancestry in Central Italian white cattle breeds overlaps with the Podolian phenotypic features we can identify in such breeds: grey coat colour, long horns, and overall rusticity. Indeed, most of these traits are shared with the Central Italian white cattle breeds; moreover, recent findings based on mitochondrial analysis identified the genetic proximity of the Italian white cattle breeds with the Turkish grey, a Podolian cattle breed from Anatolia^22^. We tested the two migration hypotheses using genotype data of seven cattle populations. Although the haplotype-based analyses we utilised (e.g., PCAdmix) are fairly robust to small sample size (Table 1), further investigations using a larger number of individuals will be necessary to test and validate our results and provide a deeper level of detail on the underlying migration scenario. We predict that the availability of genome-wide SNPs from Turkish grey cattle would allow for greater detail comparing shared zebu and African cattle ancestry with Central Italian white cattle. Nevertheless, our Admixture results identified levels of African-derived ancestry component in the Italian breeds, in accordance with previous findings^9,15,21^.

### Conclusions and outlook

Here we combine local ancestry analysis and the CIWI framework and provide the first evidence for adaptive introgression of alleles of indicine-derived ancestry into Central Italian white cattle breeds. Selective advantage of this introgression appears to result from improved feed efficiency and body size. We identified genes which might become target of ad-hoc physiological studies and targets for selection (and potentially gene editing), and eventually contribute to reduced production cost, as well as environmental burden in livestock farming (e.g., lower greenhouse gas production). Finally, for the first time, we used local ancestry information as a genomic fingerprint tool to discriminate among several migration scenarios and provided novel support for the hypothesis of multiple historical cattle migration events into the Italian peninsula.

## Supplementary information

Supplementary Table

Supplementary Figure S3

Supplementary Figure S4

Supplementary Figure S5

Supplementary Figure S6

Supplementary Figure S1a

Supplementary Figure S1b

Supplementary Figure S2

## Data Availability

All the genomic data produced in this work are available from the Mendeley Data Repository: https://dx.doi.org/10.17632/znr5dy4x29.1.
