# Supplementary material for: Adaptive introgression from indicine cattle into white cattle breeds from Central Italy
Source: Sci Rep. 2020 Jan 28;10:1279. doi: 10.1038/s41598-020-57880-4 (PMC6987186; doi:10.1038/s41598-020-57880-4)

Figure S3. Comparison of local ancestry results for BTA18 (A) and BTA7 (B) in the Chianina breed, obtained from different sets of reference populations. The analysis was performed using Hereford, Brown Swiss, and Fleckvieh (black solid line), and 25 African taurine N'Dama individuals (red dotted line) as reference for taurine cattle; in both cases Tharparkar, Gir, and Lohani were used as reference for indicine cattle. The grey shaded area denotes CW-18 (A) and CW-7 (B), respectively.

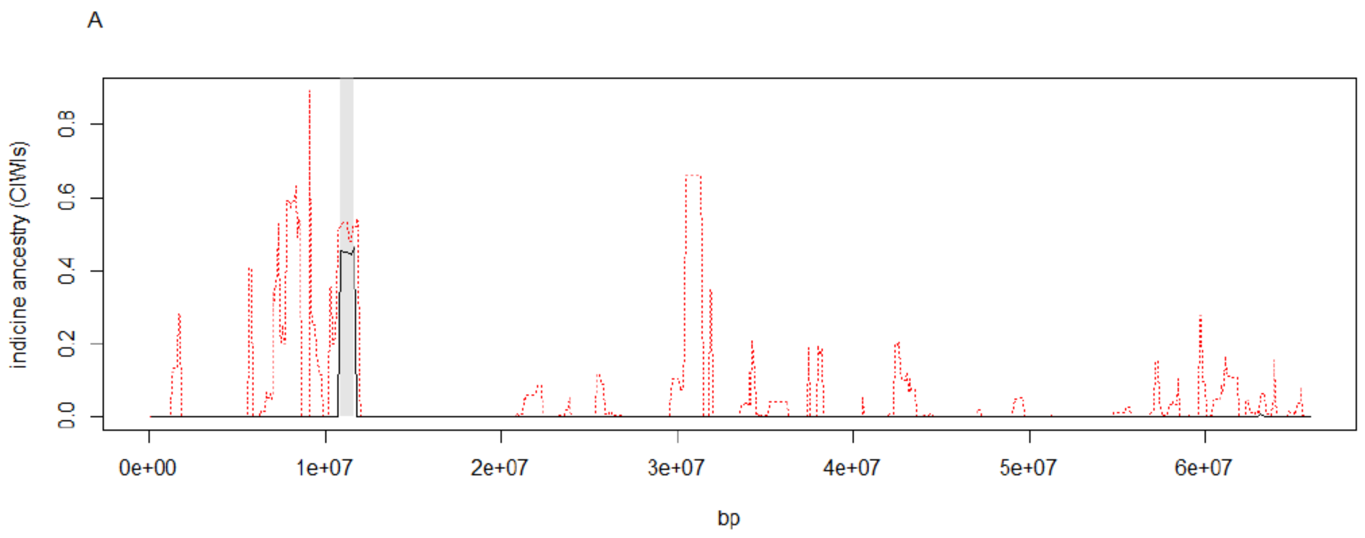

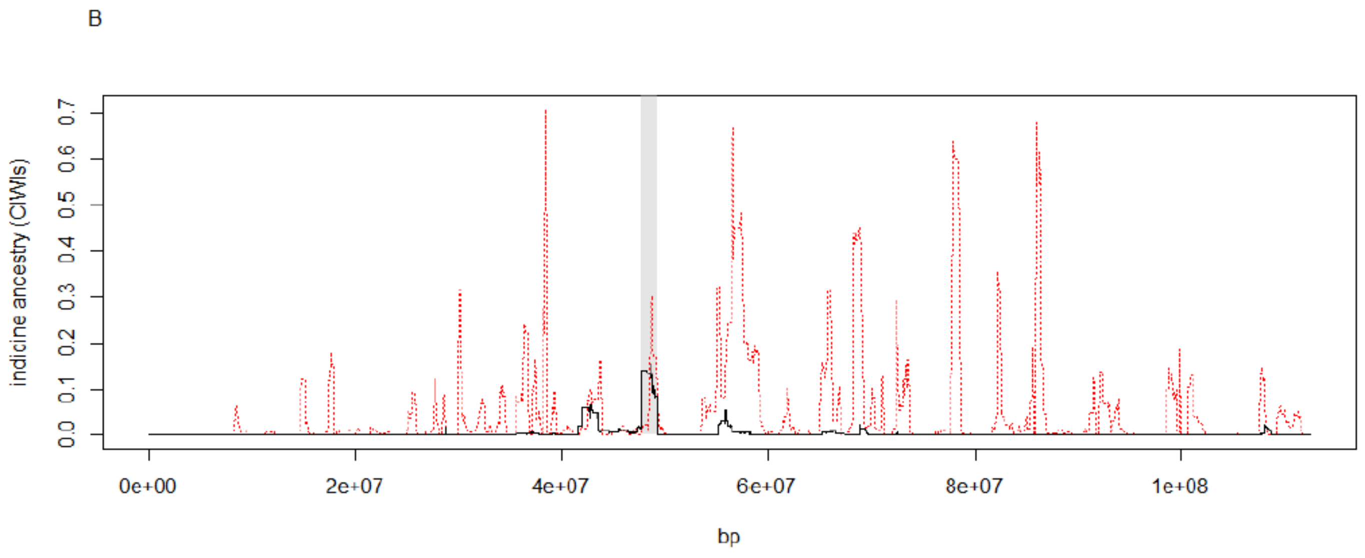

Supplement: Supplementary file 2 — Supplementary Figure S3 [file 41598_2020_57880_MOESM2_ESM.pdf]
