# Supplementary material for: Adaptive introgression from indicine cattle into white cattle breeds from Central Italy
Source: Sci Rep. 2020 Jan 28;10:1279. doi: 10.1038/s41598-020-57880-4 (PMC6987186; doi:10.1038/s41598-020-57880-4)

Figure S4. Selection analysis performed on the CHI chromosomes where CIWIs were identified. The solid black line represents the cubic smoothed spline of the |nSL| selection signals. The grey shading denotes CIWIs.

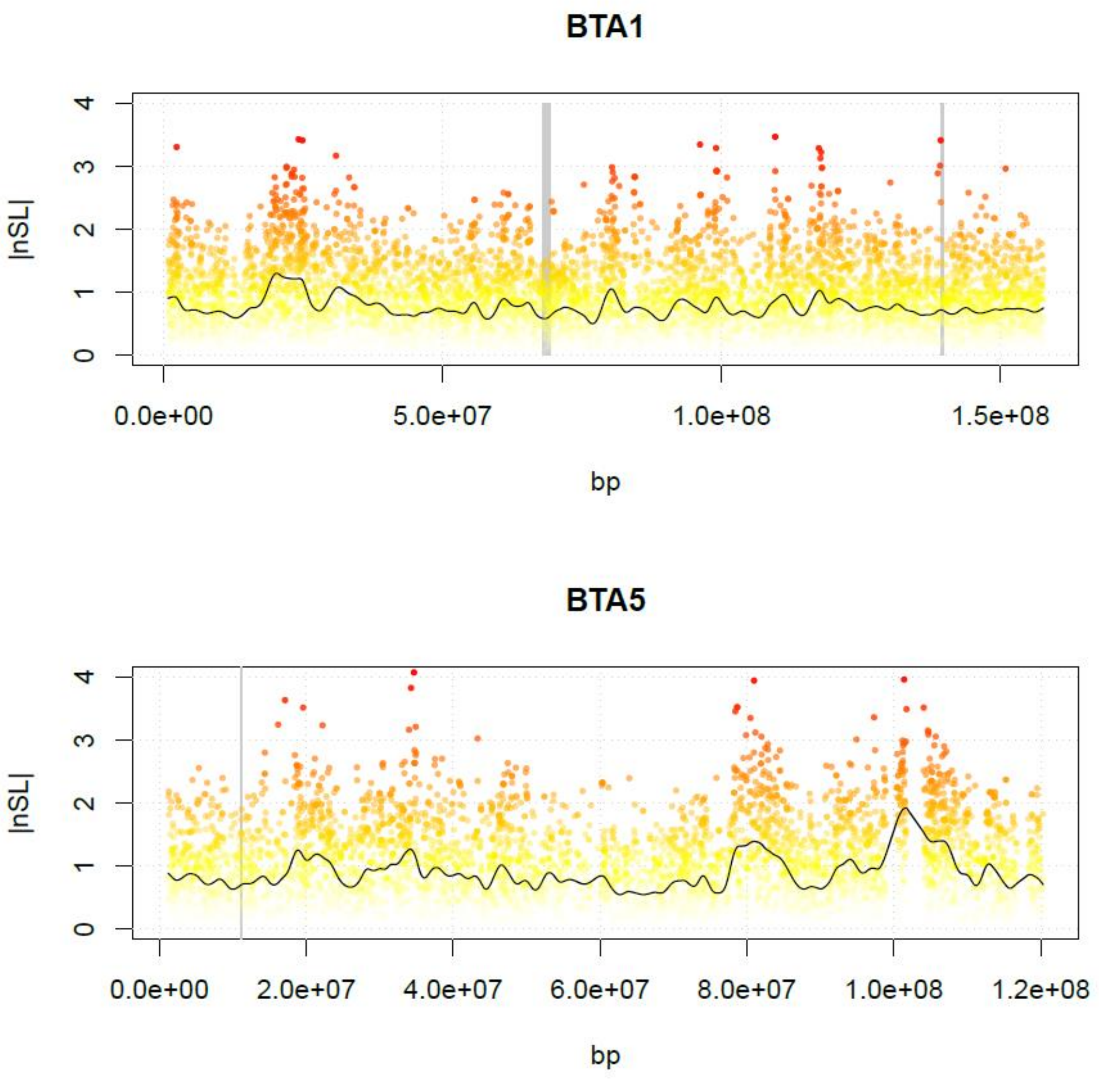

Supplement: Supplementary file 3 — Supplementary Figure S4 [file 41598_2020_57880_MOESM3_ESM.pdf]
