# Supplementary material for: Adaptive introgression from indicine cattle into white cattle breeds from Central Italy
Source: Sci Rep. 2020 Jan 28;10:1279. doi: 10.1038/s41598-020-57880-4 (PMC6987186; doi:10.1038/s41598-020-57880-4)
Supplement: Supplementary file 4 — Supplementary Figure S5 [file 41598_2020_57880_MOESM4_ESM.pdf]

Figure S5. CIWI results for 29 chromosomes. Chianina was used as target population, Hereford, Fleckvieh and Brown Swiss as taurine references, and Lohani, Gir and Tharparkar as indicine references. The solid black line represents the score assigned to the CIWI of indicine ancestry. See Table 1 for population labels.

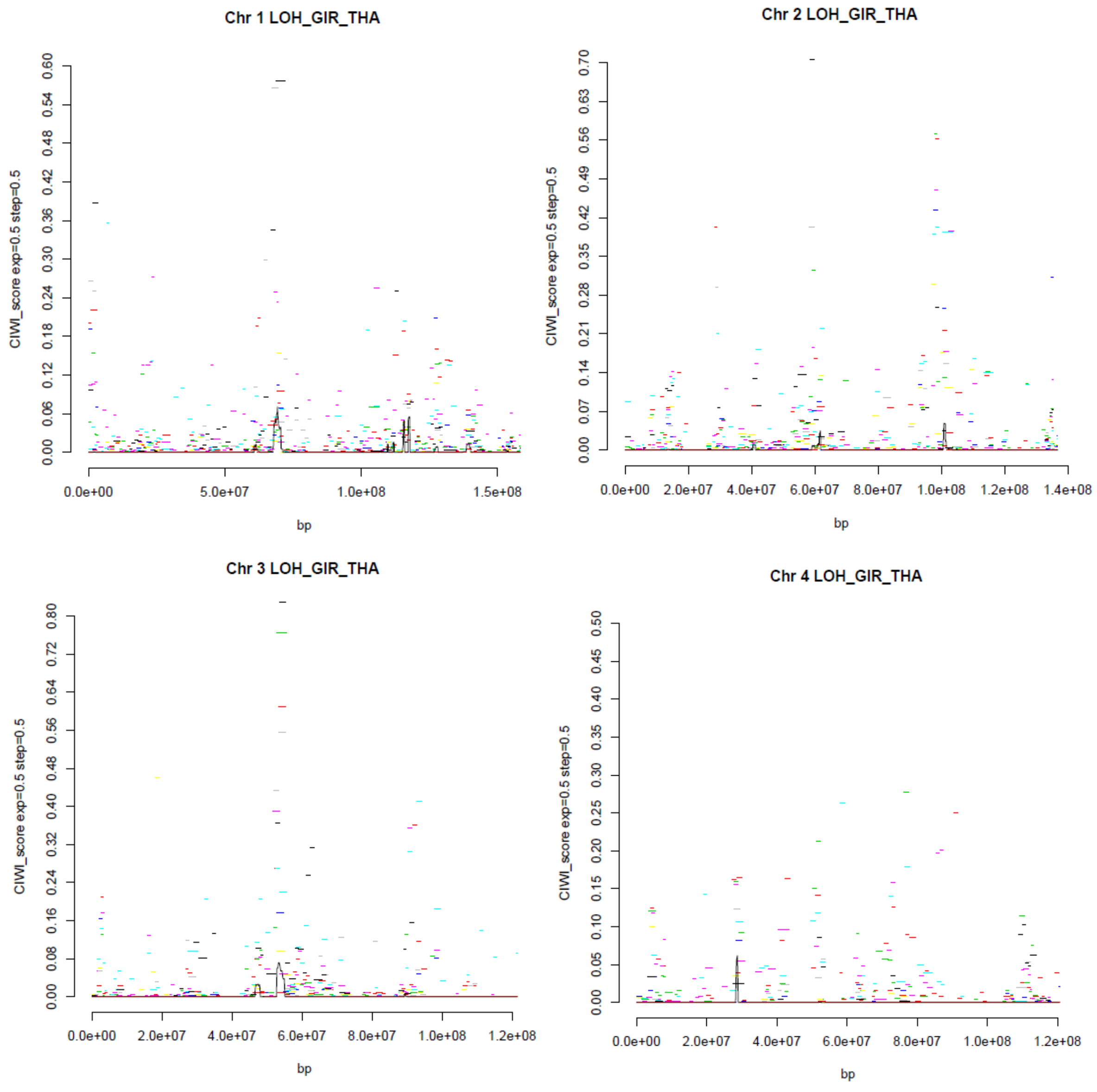

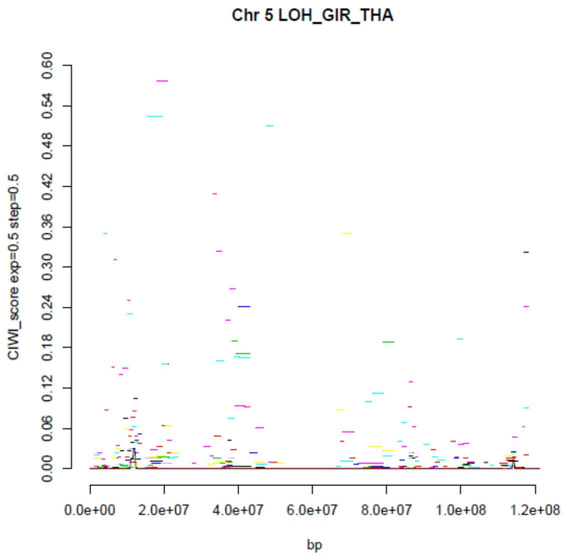
Chr 5 LOH_GIR_THA
CIWI_score exp=0.5 step=0.5
0.00 0.06 0.12 0.18 0.24 0.30 0.36 0.42 0.48 0.54 0.60
0.0e+00 2.0e+07 4.0e+07 6.0e+07 8.0e+07 1.0e+08 1.2e+08
bp

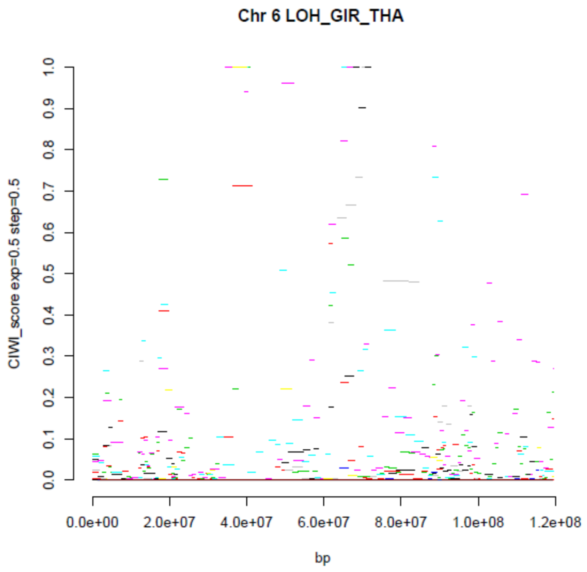
Chr 6 LOH_GIR_THA
CIWI_score exp=0.5 step=0.5
0.0 0.1 0.2 0.3 0.4 0.5 0.6 0.7 0.8 0.9 1.0
0.0e+00 2.0e+07 4.0e+07 6.0e+07 8.0e+07 1.0e+08 1.2e+08
bp

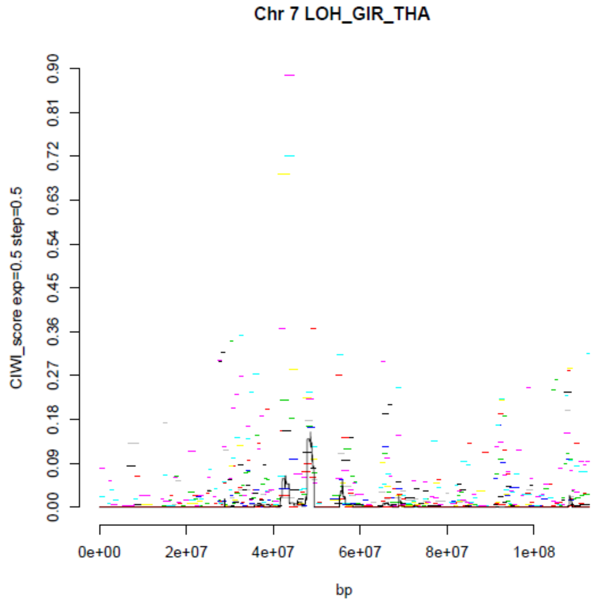
Chr 7 LOH_GIR_THA
CIWI_score exp=0.5 step=0.5
0.00 0.09 0.18 0.27 0.36 0.45 0.54 0.63 0.72 0.81 0.90
0e+00 2e+07 4e+07 6e+07 8e+07 1e+08
bp

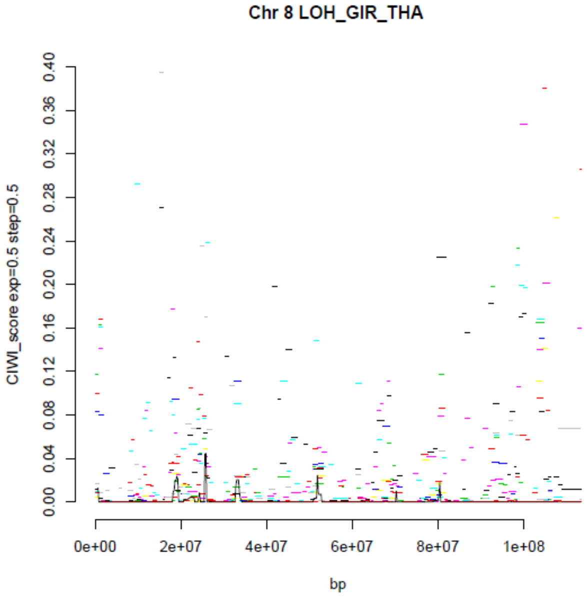
Chr 8 LOH_GIR_THA
CIWI_score exp=0.5 step=0.5
0.00 0.04 0.08 0.12 0.16 0.20 0.24 0.28 0.32 0.36 0.40
0e+00 2e+07 4e+07 6e+07 8e+07 1e+08
bp

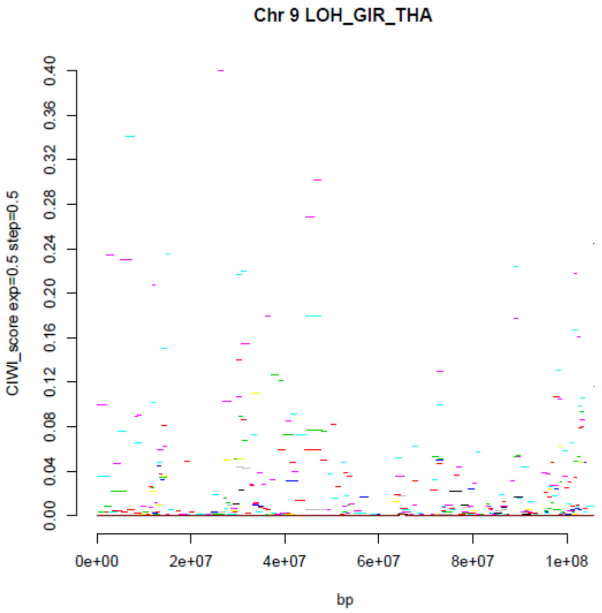
Chr 9 LOH_GIR_THA
CIWI_score exp=0.5 step=0.5
0.00 0.04 0.08 0.12 0.16 0.20 0.24 0.28 0.32 0.36 0.40
0e+00 2e+07 4e+07 6e+07 8e+07 1e+08
bp

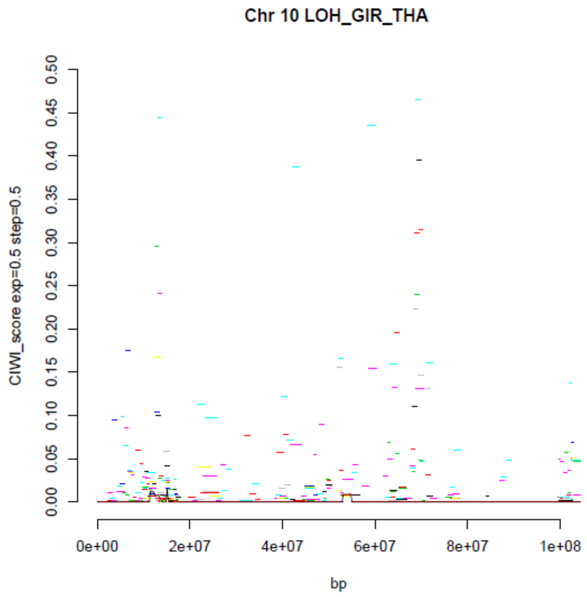
Chr 10 LOH_GIR_THA
CIWI_score exp=0.5 step=0.5
0.00 0.05 0.10 0.15 0.20 0.25 0.30 0.35 0.40 0.45 0.50
0e+00 2e+07 4e+07 6e+07 8e+07 1e+08
bp

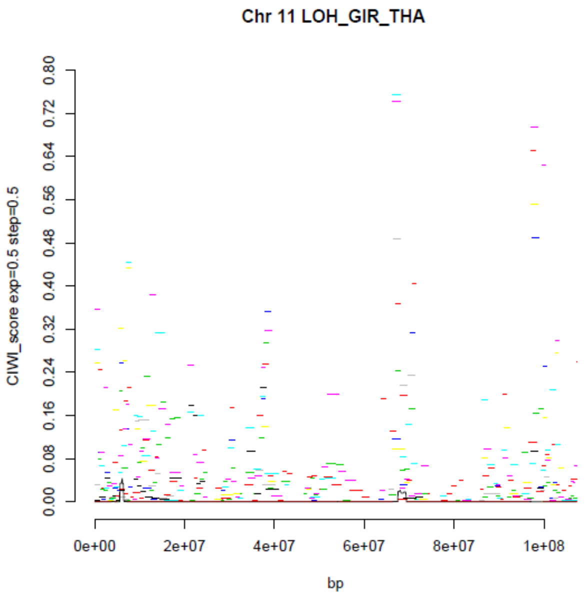
Chr 11 LOH_GIR_THA
CIWI_score exp=0.5 step=0.5
0.00 0.08 0.16 0.24 0.32 0.40 0.48 0.56 0.64 0.72 0.80
0e+00 2e+07 4e+07 6e+07 8e+07 1e+08
bp

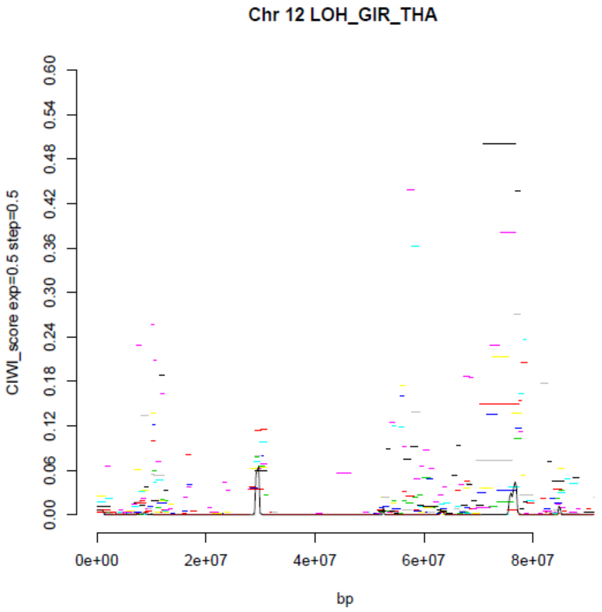
Chr 12 LOH_GIR_THA
CIWI_score exp=0.5 step=0.5
0.00 0.06 0.12 0.18 0.24 0.30 0.36 0.42 0.48 0.54 0.60
0e+00 2e+07 4e+07 6e+07 8e+07
bp

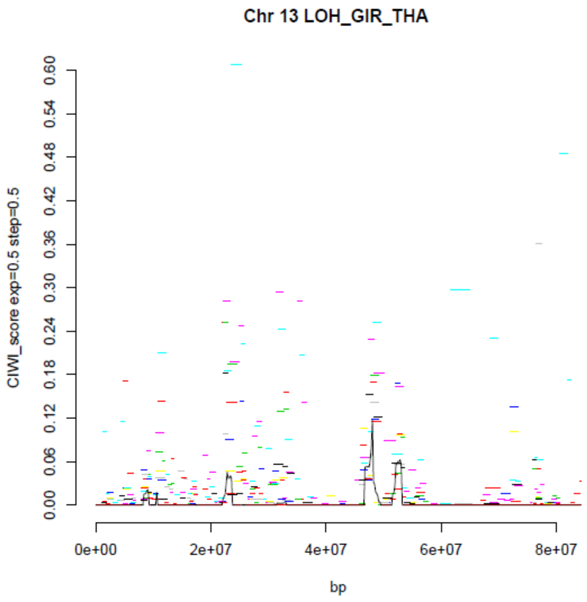
Chr 13 LOH_GIR_THA
CIWI_score exp=0.5 step=0.5
0.00 0.06 0.12 0.18 0.24 0.30 0.36 0.42 0.48 0.54 0.60
0e+00 2e+07 4e+07 6e+07 8e+07
bp

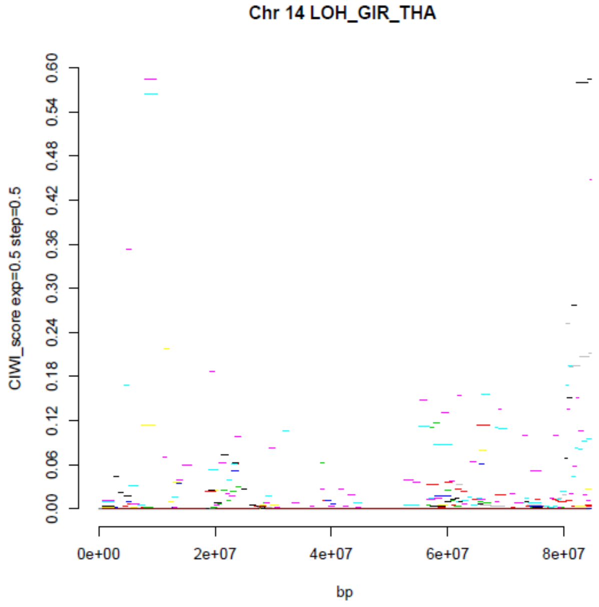
Chr 14 LOH_GIR_THA
CIWI_score exp=0.5 step=0.5
0.00 0.06 0.12 0.18 0.24 0.30 0.36 0.42 0.48 0.54 0.60
0e+00 2e+07 4e+07 6e+07 8e+07
bp

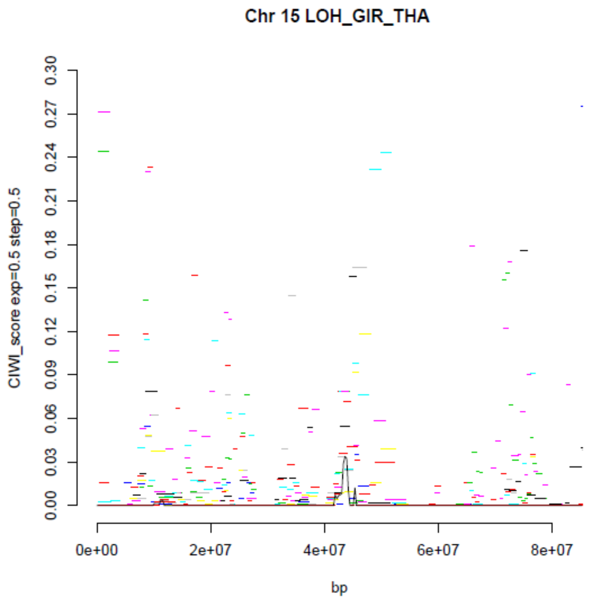
Chr 15 LOH_GIR_THA
CIWI_score exp=0.5 step=0.5
0.00 0.03 0.06 0.09 0.12 0.15 0.18 0.21 0.24 0.27 0.30
0e+00 2e+07 4e+07 6e+07 8e+07
bp

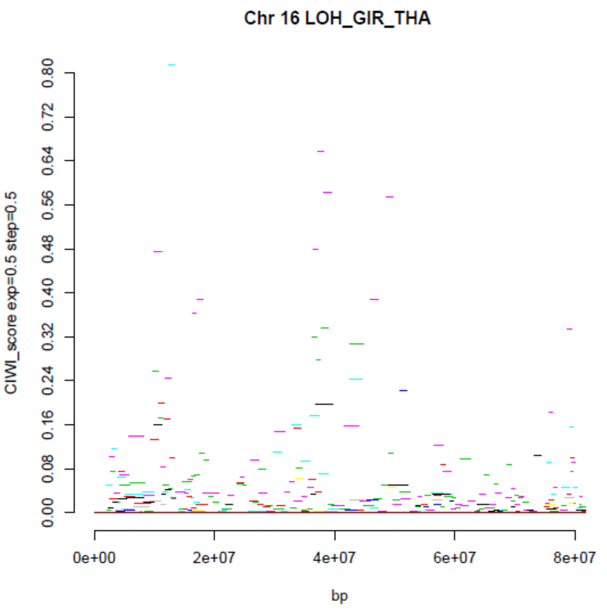
Chr 16 LOH_GIR_THA
CIWI_score exp=0.5 step=0.5
0.00 0.08 0.16 0.24 0.32 0.40 0.48 0.56 0.64 0.72 0.80
0e+00 2e+07 4e+07 6e+07 8e+07
bp

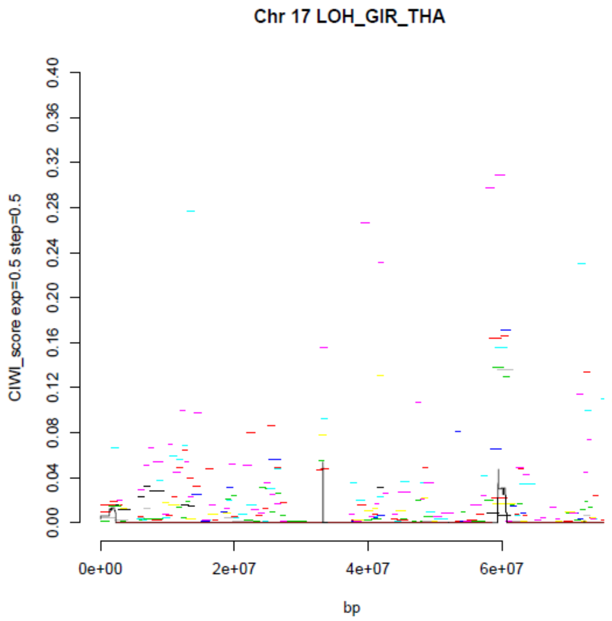
Chr 17 LOH_GIR_THA
CIWI_score exp=0.5 step=0.5
0.00 0.04 0.08 0.12 0.16 0.20 0.24 0.28 0.32 0.36 0.40
0e+00 2e+07 4e+07 6e+07
bp

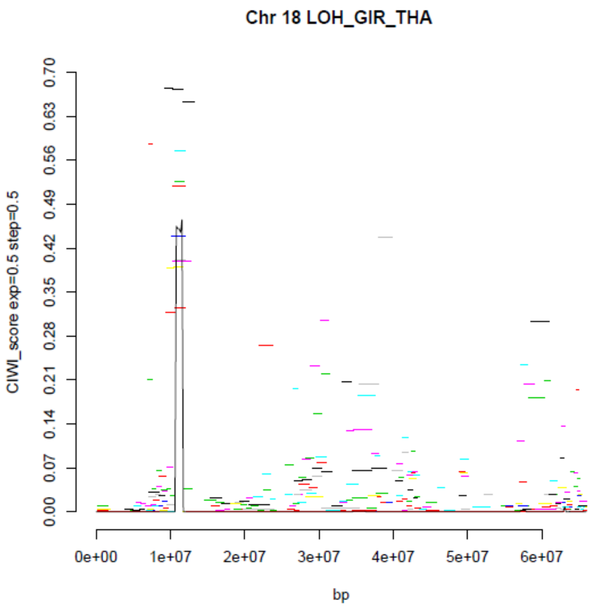
Chr 18 LOH_GIR_THA
CIWI_score exp=0.5 step=0.5
0.00 0.07 0.14 0.21 0.28 0.35 0.42 0.49 0.56 0.63 0.70
0e+00 1e+07 2e+07 3e+07 4e+07 5e+07 6e+07
bp

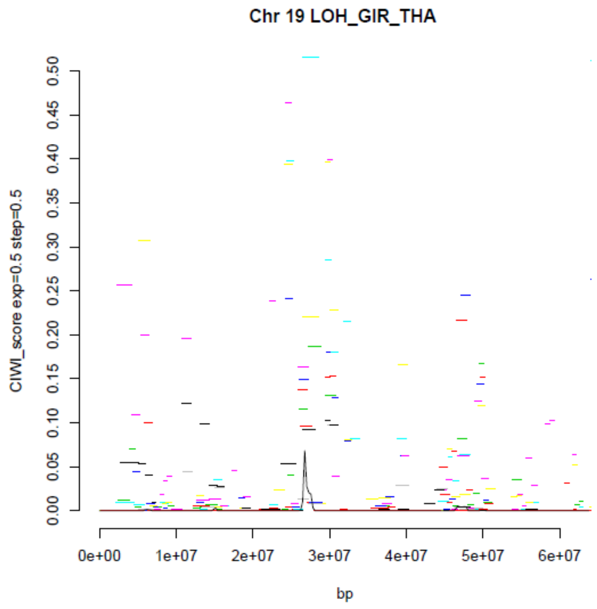
Chr 19 LOH_GIR_THA
CIWI_score exp=0.5 step=0.5
0.00 0.05 0.10 0.15 0.20 0.25 0.30 0.35 0.40 0.45 0.50
0e+00 1e+07 2e+07 3e+07 4e+07 5e+07 6e+07
bp

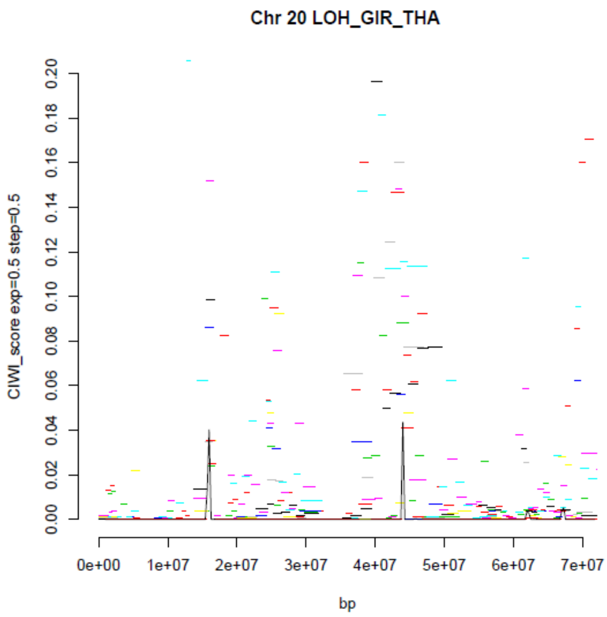
Chr 20 LOH_GIR_THA
CIWI_score exp=0.5 step=0.5
0.00 0.02 0.04 0.06 0.08 0.10 0.12 0.14 0.16 0.18 0.20
0e+00 1e+07 2e+07 3e+07 4e+07 5e+07 6e+07 7e+07
bp

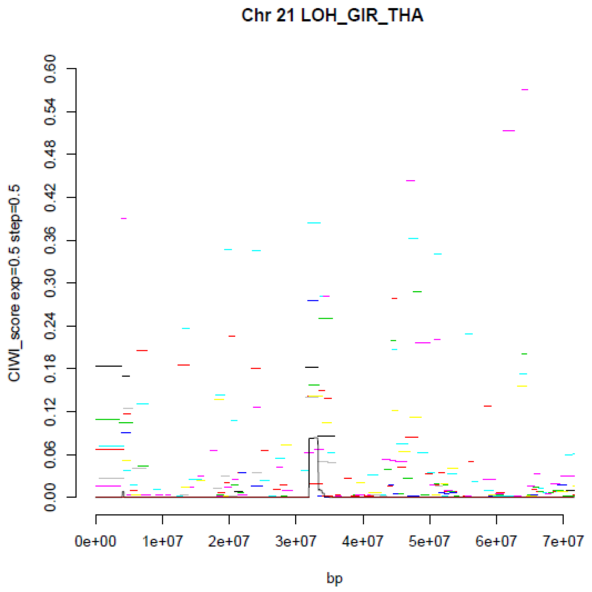

Chr 21 LOH_GIR_THA
CIWI_score exp=0.5 step=0.5
0.00 0.06 0.12 0.18 0.24 0.30 0.36 0.42 0.48 0.54 0.60
0e+00 1e+07 2e+07 3e+07 4e+07 5e+07 6e+07 7e+07
bp

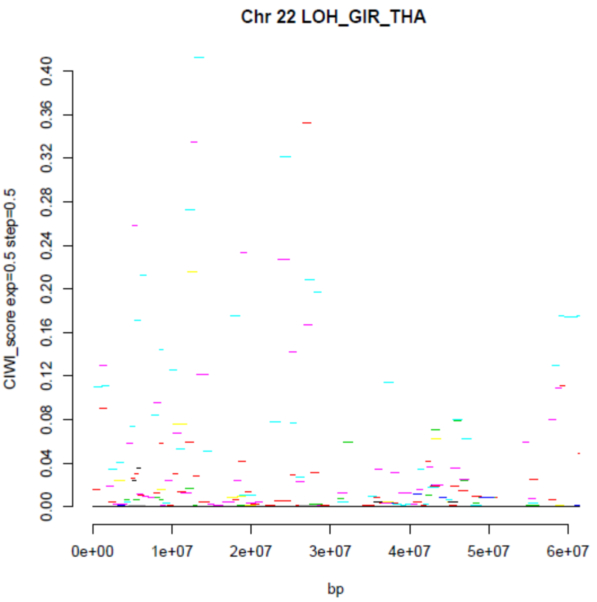

Chr 22 LOH_GIR_THA
CIWI_score exp=0.5 step=0.5
0.00 0.04 0.08 0.12 0.16 0.20 0.24 0.28 0.32 0.36 0.40
0e+00 1e+07 2e+07 3e+07 4e+07 5e+07 6e+07
bp

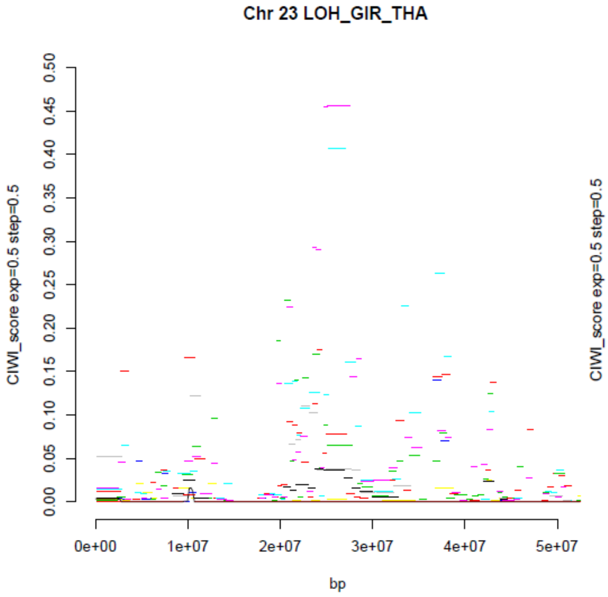

Chr 23 LOH_GIR_THA
CIWI_score exp=0.5 step=0.5
0.00 0.05 0.10 0.15 0.20 0.25 0.30 0.35 0.40 0.45 0.50
0e+00 1e+07 2e+07 3e+07 4e+07 5e+07
bp

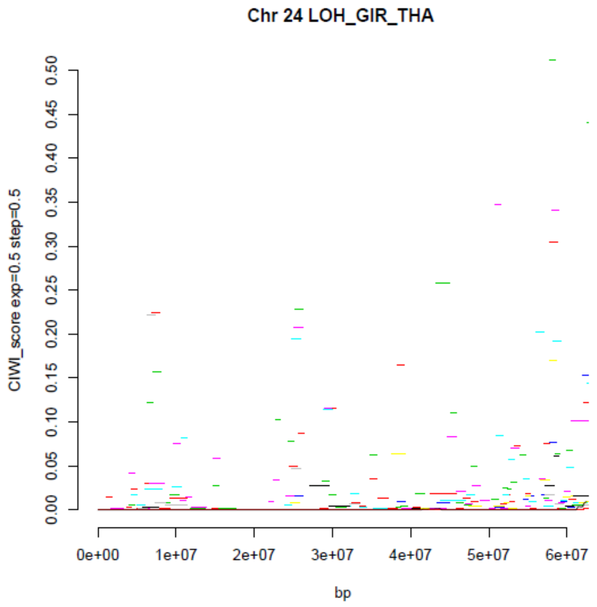

Chr 24 LOH_GIR_THA
CIWI_score exp=0.5 step=0.5
0.00 0.05 0.10 0.15 0.20 0.25 0.30 0.35 0.40 0.45 0.50
0e+00 1e+07 2e+07 3e+07 4e+07 5e+07 6e+07
bp

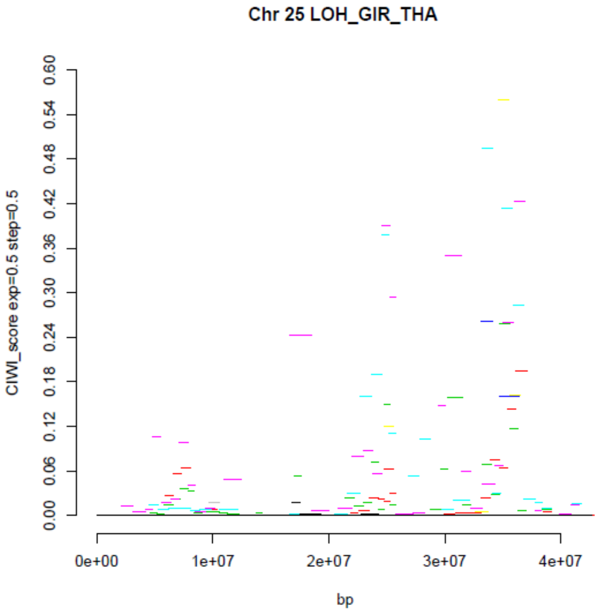

Chr 25 LOH_GIR_THA
CIWI_score exp=0.5 step=0.5
0.00 0.06 0.12 0.18 0.24 0.30 0.36 0.42 0.48 0.54 0.60
0e+00 1e+07 2e+07 3e+07 4e+07
bp

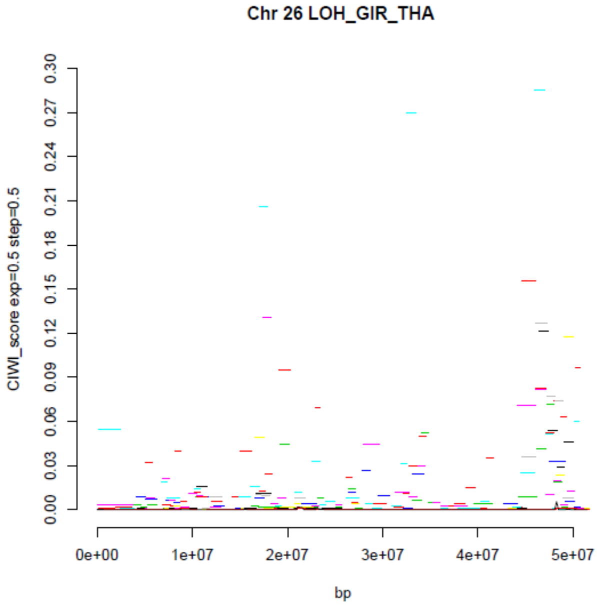

Chr 26 LOH_GIR_THA
CIWI_score exp=0.5 step=0.5
0.00 0.03 0.06 0.09 0.12 0.15 0.18 0.21 0.24 0.27 0.30
0e+00 1e+07 2e+07 3e+07 4e+07 5e+07
bp

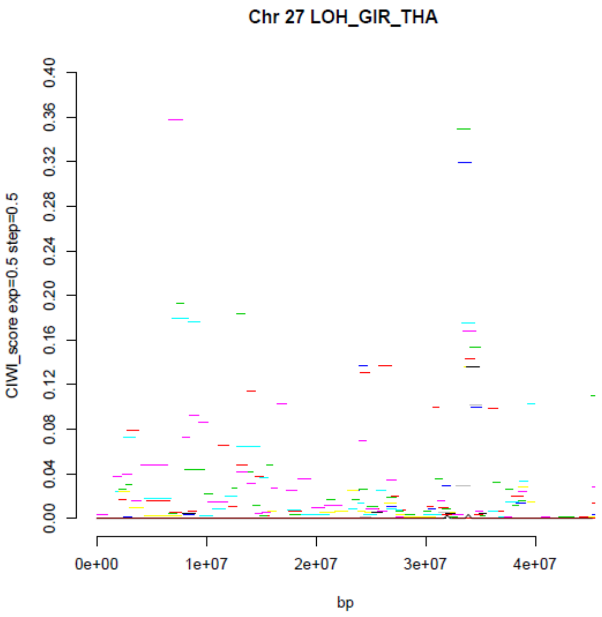
Chr 27 LOH_GIR_THA
CIWI_score exp=0.5 step=0.5
0.00
0.04
0.08
0.12
0.16
0.20
0.24
0.28
0.32
0.36
0.40
0e+00
1e+07
2e+07
3e+07
4e+07
bp

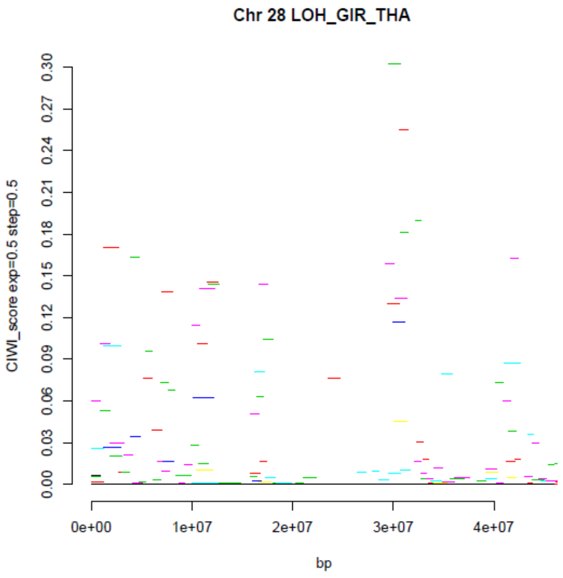
Chr 28 LOH_GIR_THA
CIWI_score exp=0.5 step=0.5
0.00
0.03
0.06
0.09
0.12
0.15
0.18
0.21
0.24
0.27
0.30
0e+00
1e+07
2e+07
3e+07
4e+07
bp

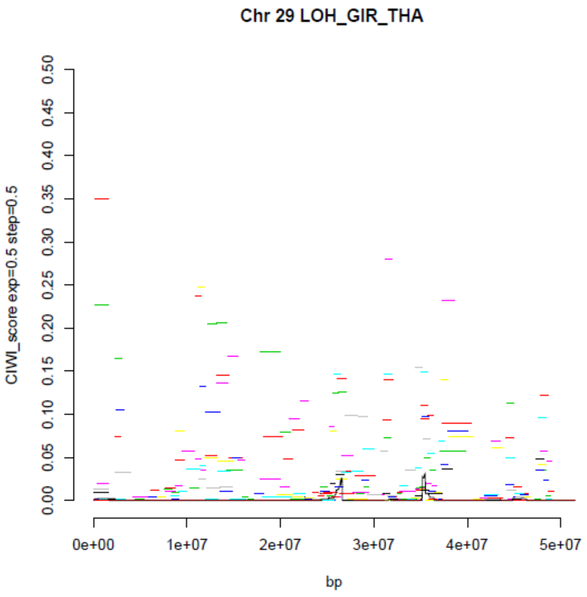
Chr 29 LOH_GIR_THA
CIWI_score exp=0.5 step=0.5
0.00
0.05
0.10
0.15
0.20
0.25
0.30
0.35
0.40
0.45
0.50
0e+00
1e+07
2e+07
3e+07
4e+07
5e+07
bp
