# Supplementary material for: Adaptive introgression from indicine cattle into white cattle breeds from Central Italy
Source: Sci Rep. 2020 Jan 28;10:1279. doi: 10.1038/s41598-020-57880-4 (PMC6987186; doi:10.1038/s41598-020-57880-4)

Figure S6. PCA plots of 16 cattle breeds based on 647,132 SNPs from the BovineHD SNPChip. Value in brackets refer to the proportion of variance explained by each PC. Breed labels are available in Table 1.

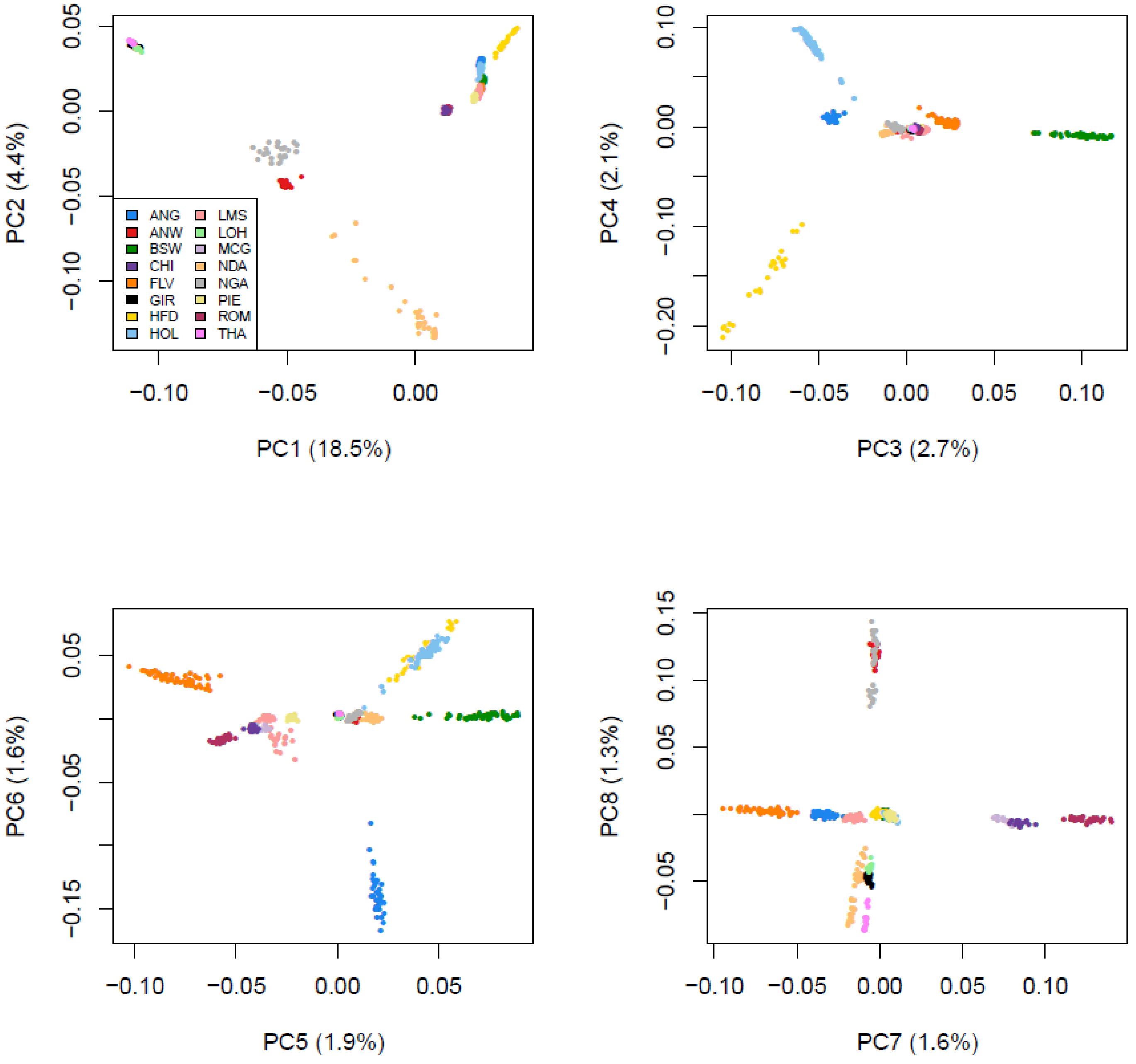

Supplement: Supplementary file 5 — Supplementary Figure S6 [file 41598_2020_57880_MOESM5_ESM.pdf]
