# Supplementary material for: Adaptive introgression from indicine cattle into white cattle breeds from Central Italy
Source: Sci Rep. 2020 Jan 28;10:1279. doi: 10.1038/s41598-020-57880-4 (PMC6987186; doi:10.1038/s41598-020-57880-4)

Figure S1a. Admixture plot of 16 cattle breeds based on 647,132 SNPs from the BovineHD SNPChip. Breed labels are available in Table 1.

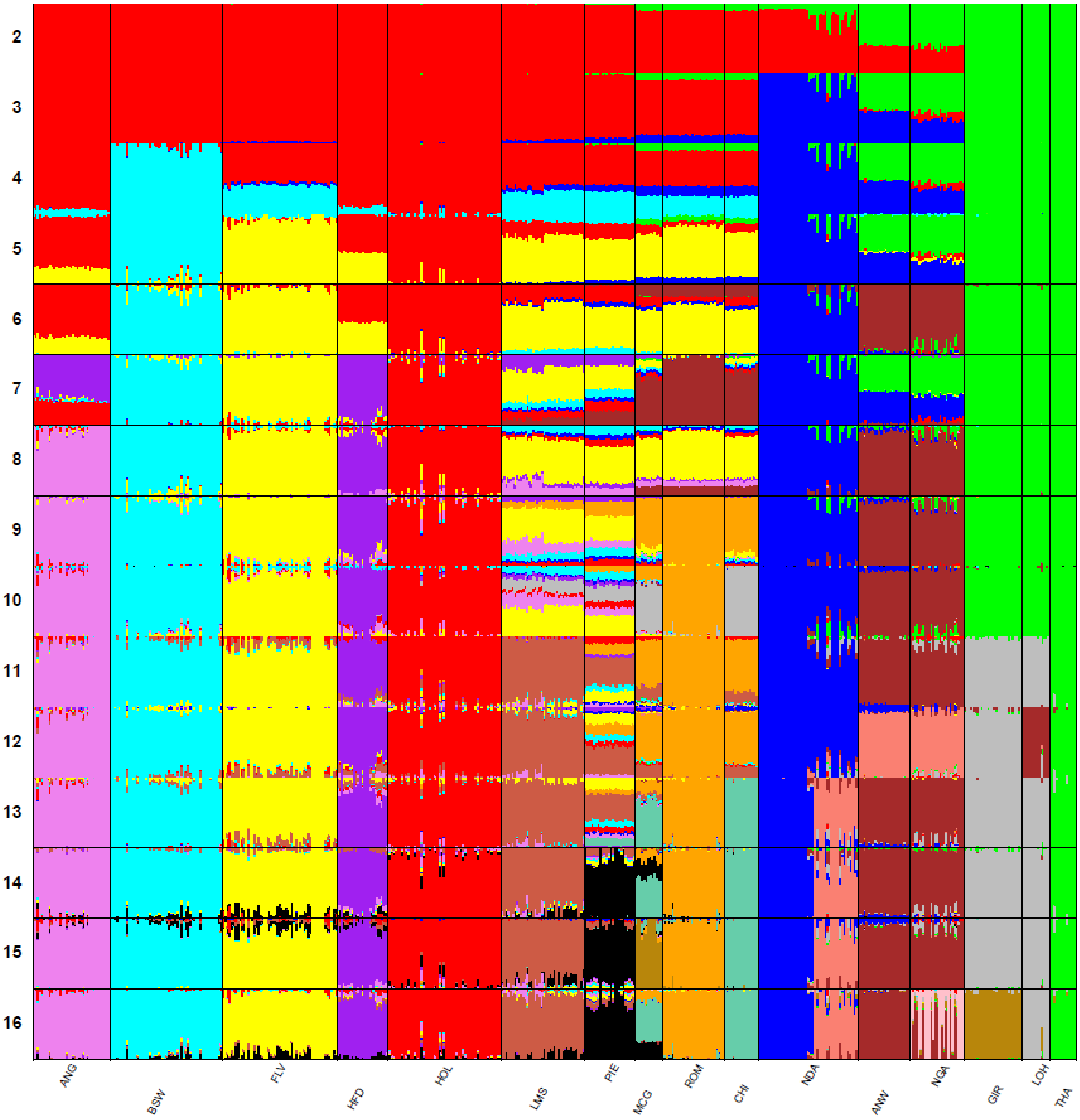

Supplement: Supplementary file 6 — Supplementary Figure S1a [file 41598_2020_57880_MOESM6_ESM.pdf]
