# Supplementary figures and images for: Adaptive introgression from indicine cattle into white cattle breeds from Central Italy
Source: Sci Rep. 2020 Jan 28;10:1279. doi: 10.1038/s41598-020-57880-4 (PMC6987186; doi:10.1038/s41598-020-57880-4)

Figure S1b. Cross validation error calculated for each value of  $K = 1-16$ .

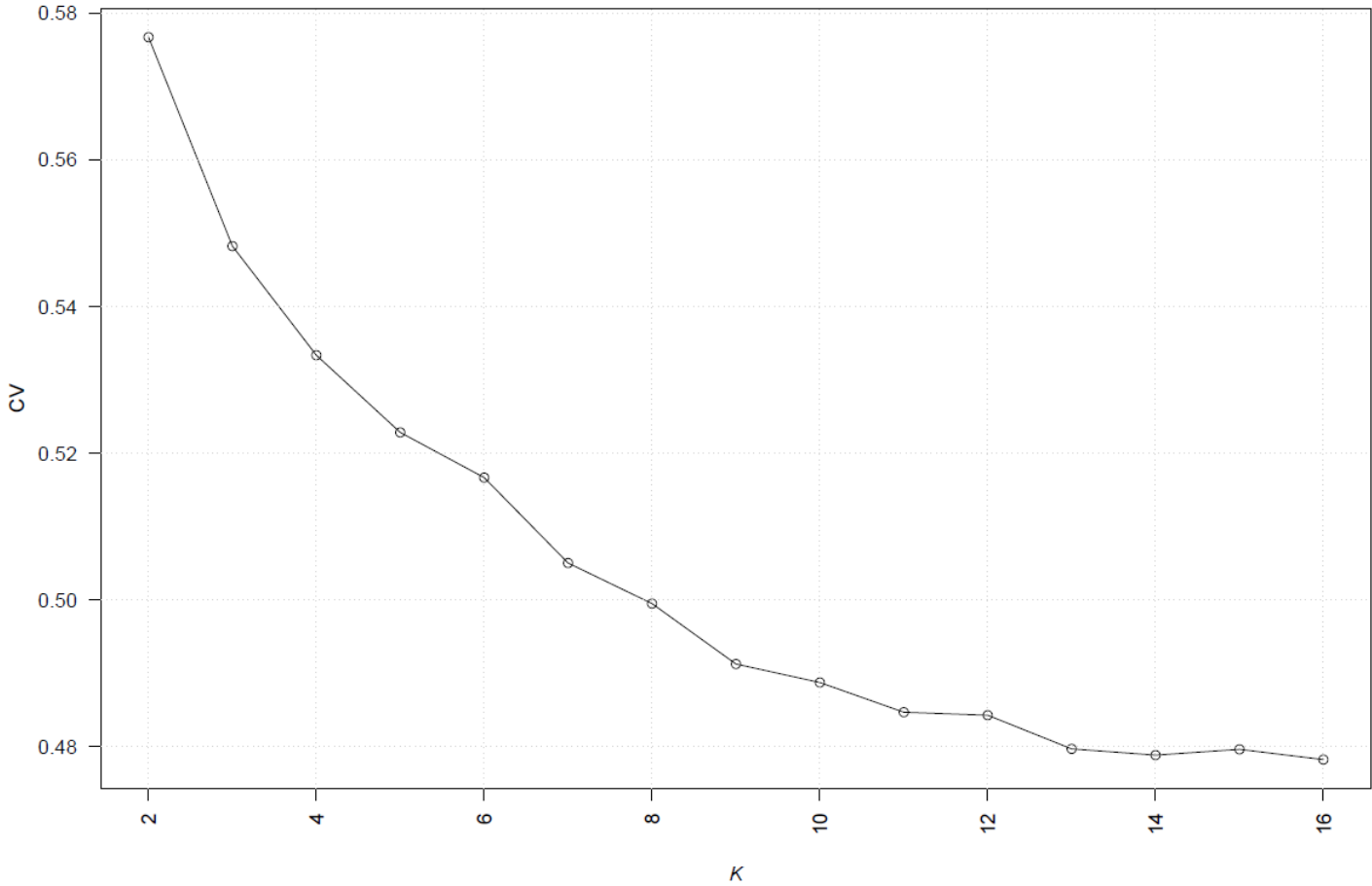

Supplement: Supplementary file 7 — Supplementary Figure S1b [file 41598_2020_57880_MOESM7_ESM.pdf]
