# Supplementary material for: Adaptive introgression from indicine cattle into white cattle breeds from Central Italy
Source: Sci Rep. 2020 Jan 28;10:1279. doi: 10.1038/s41598-020-57880-4 (PMC6987186; doi:10.1038/s41598-020-57880-4)

Figure S2. Neighbour-Net network computed using a matrix of Reynold's distances between 16 cattle breeds, based on BovineHD data. Inner branches proportions are modified through the 'magnify' tool in TreeView for improved readability. Colours refer to the geographical origin of the breeds: European (red), African (blue), and Indian (green). Breed labels are defined in Table 1.

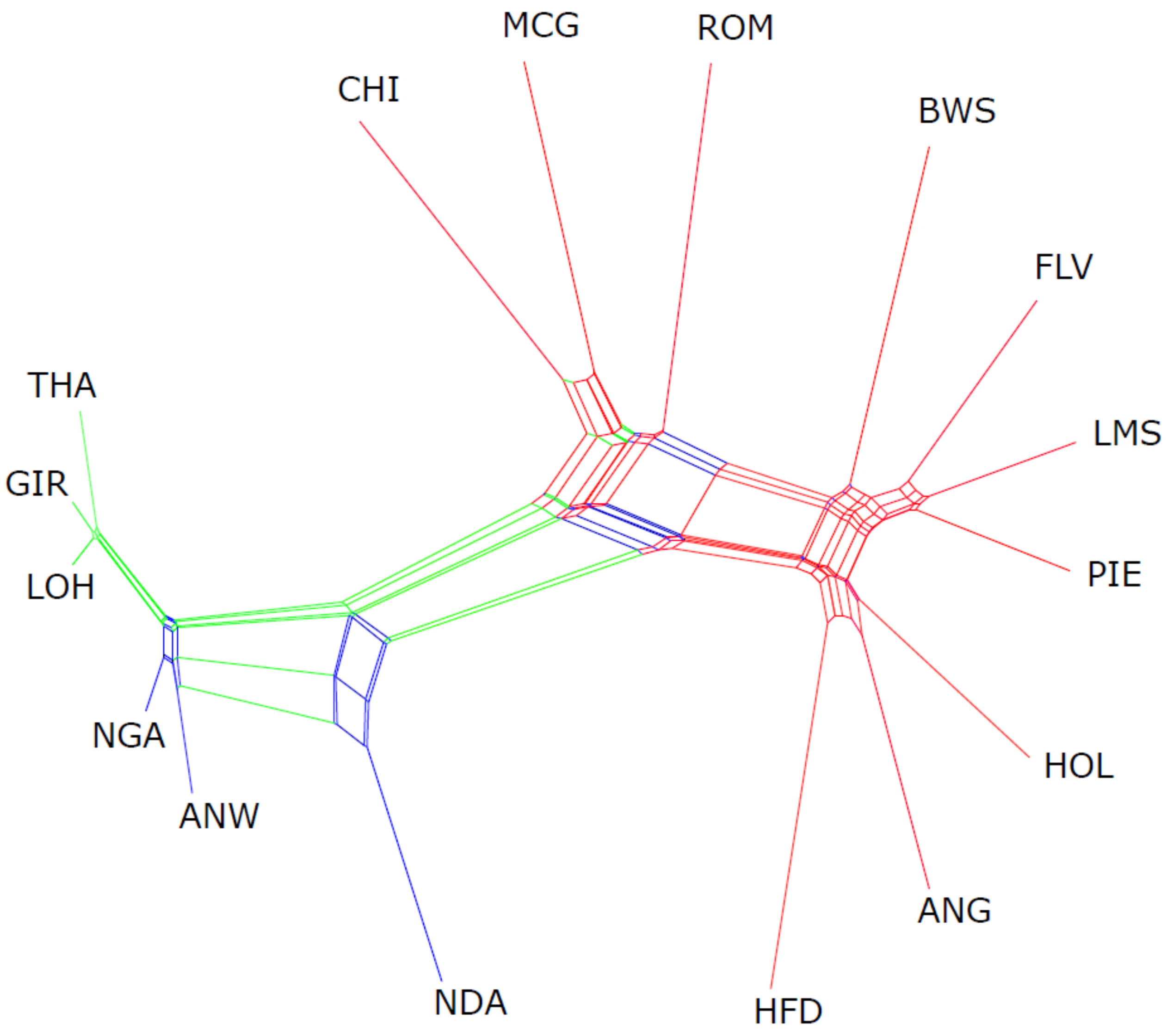

Supplement: Supplementary file 8 — Supplementary Figure S2 [file 41598_2020_57880_MOESM8_ESM.pdf]
